# Upper and Lower Face and Ideomotor Apraxia in Patients with Alzheimer’s Disease

**DOI:** 10.1155/2003/518959

**Published:** 2003-01-01

**Authors:** Jay Guido Capone, Sergio Della Sala, Hans Spinnler, Annalena Venneri

**Affiliations:** ^1^Third Neurology WardSan Paolo HospitalDepartment of MedicineSurgery and DentistryUniversity of MilanMilanItaly; ^2^Neuropsychology Research GroupDepartment of PsychologyUniversity of AberdeenUK

**Keywords:** face apraxia, ideomotor apraxia, Alzheimer’s disease

## Abstract

*Introduction:* Apraxia of face movement in Alzheimer's disease (AD) has been rarely investigated. This study aimed at investigating the frequency of lower (mouth, tongue and throat) and upper (eyes and eyebrows) face apraxia, in AD and its relationship with limb apraxia and severity of dementia.

*Methods:* Fifty seven patients with AD were tested with a new standardised test of face apraxia including upper and lower face movements, which uses an item-difficulty weigthed scoring procedure, the IMA test, a test of ideomotor apraxia and the M.O.D.A., a means to assess dementia severity.

*Results:* Thirteen (23%) and 19 (33%) participants were below cut-off respectively on the upper and lower face apraxia test. Both sections of the Face Apraxia Test correlated significantly with the Ideomotor Apraxia Test. However, double dissociations between different types of apraxia were observed. Both the upper and lower face apraxia tests correlated significantly with the measure of dementia severity.

*Conclusions:* The finding show that a proportion of AD patients fails face apraxia tests. Their face apraxia is interlinked with ideomotor limb apraxia, although dissociations are possible. Severity of dementia deterioration accounts for a good proportion of the variability of AD patients’ performance on face apraxia tests.

## 1. Introduction

Apraxia generally refers to the impaired ability to perform movements upon request [56]. This impairment should not be accountable for in terms of impairment of motor or sensory functions [13]. The term apraxia usually refers to a variety of disorders which are specified more in detail either referring to the regions of the body affected, or the testing procedures employed or the theoretical framework of reference adopted. Several group studies have reported ideomotor limb apraxia or ideational apraxia in patients with Alzheimer's disease (AD) [20,25,39,69] (review in [57]). Apraxia of face movements in patients with (AD) has, however, received very little attention and its frequency and characteristics have yet to be defined in this patient population.

Face apraxia involves intentional movements of the face muscles [14]. It should be kept separate from "apraxia of speech" [60] and "pure anarthria" [41]; both these disorders result from disturbances of word articulation rather than of oro-facial movements (see reviews in [36] and [59]).

Generally, authors refer to face apraxia as oral apraxia, because, in most instances, only the movements of the lower face are examined [17,38,58]. Several papers, however, reported patients with oral apraxia following focal brain lesions who also showed deficits of the movements of the upper part of the face (eyes and eyebrows) (e.g. [12,36,37,50]).

Although face apraxia is probably the most frequent of all apraxias due to focal brain lesions [53], it is sel-

ISSN 0953-4180/03/$8.00 © 2003 – IOS Press. All rights reserved dom assessed in clinical practice and very few controlled experimental studies have been carried out. The dearth of experimental investigations on face apraxia is even more noticeable when one searches for studies of face apraxia in patients with degenerative brain diseases. Face apraxia, either of the lower (e.g. [2,12,47, 65,66]) or the upper face (e.g. [1,35]), has been mentioned in passing in relation to speech impairment in a few cases of patients affected by non-Alzheimer type dementias.

Only a few studies are available in the literature in which face apraxia is reported in AD. Marcuse [40] reported the case of a patient, W., a 60-year old woman with a history of 3–4 years of progressive memory and cognitive deterioration with amnesiac onset, without any apparent neurological sign. This patient showed lower and upper face apraxia; for example, she repeatedly failed to stick out her tongue, wrinkle her forehead or direct her gaze on the horizontal plane on verbal command. Perea and Ladera [48] tested 54 patients with AD and reported that their poor performance on a scale measuring activities of daily living could be partly accounted for by their low scores on a short test assessing oral apraxia. Kokmen et al. [33] reported findings of a Turkish/American cross-cultural validation study on an apraxia test, which included ten items for the assessment of movements of the lower face, in which responses were scored using a pass/fail criterion (score 1-0). These authors tested also 18 AD patients whose mean score on the oral apraxia items was significantly poorer than that of a group of healthy controls. Rapcsak et al. [55] and Taylor [62] carried out two independent studies whose main focus was to investigate the frequency of lower face apraxia in AD. In both studies a 5-item test was used. Data from these studies yielded fairly contrasting results: Rapcsak et al. [55] stated that less than 20% of AD patients were affected by face apraxia, while Taylor [62] maintained that face apraxia was present in over 90% of the AD patients studied.

Therefore, data on the frequency of face apraxia in AD are scant and contrasting. Further, issues related to the relationship between face apraxia and dementia severity remain unsettled. Moreover, upper face apraxia has never been investigated in patients with AD. The relationship between limb and face apraxia in patients with focal brain lesions has been matter for debate. Some authors supported a unitary mechanism underlying both deficits, (e.g. [51]), while others maintained that these deficits result from partially independent mechanisms (e.g. [6]) or that they may dissociate completely (e.g. [17,54]). Ideomotor and face apraxia, however, were never directly compared in patients with AD.

Studies with AD patients have already made a substantial empirical contribution to cognitive models in several domains - such as working memory [3,7] face processing [21], language [19,49], visual perception [18,22] and semantic memory [4,29], distribution of attention in space [5,67]. This contribution has added to our understanding of cognitive systems and their anatomical substrates and stems from the uniqueness of the Alzheimer neuropathology that is characterised by a fine grained and diffuse progressive damage of cerebral networks. Traditionally face apraxia has been linked to lesions in the left hemisphere and associated with non-fluent aphasia [17,46,52,61], probably because of anatomical contiguity [13,63]. However, more recent findings suggest that damage to a much broader neuronal network, involving both the left and the right hemisphere, may result in lower and upper face apraxia [9]. Therefore, patients with AD, who suffer from a widespread and slowly progressive degeneration are ideal candidates for verifying the hypothesis that a larger network is responsible for face eupraxia.

The aim of this study was to investigate the frequency of lower (mouth, tongue and throat) and upper (eyes and eyebrows) face apraxia in patients with AD using a new standardized test, for which norms collected on a sample of 180 healthy controls are available [23]. This test has also been validated on a group of stroke patients [9,23]. A further aim of this study was to clarify the relationship between face apraxia, limb apraxia and severity of dementia as well as verify whether dissociating performances on the two types of apraxia test are present.

## 2. Methods

### 2.1. Participants

Fifty seven out-patients (14 male, 43 female) meeting NINCDS-ADRDA clinical criteria for possible or probable AD [43] were included in the study. These patients were recruited from referrals to the Dementia Neuropsychology Unit of the Third Neurology Clinic, San Paolo Hospital, University of Milan. The age of onset in 15% of the cases was below 70. A CT (or MRI) brain scan was performed on all the patients. Age in this sample ranged from 51 to 88 years (mean 72.85, SD 8.48). The education range was 1-19 years (mean 7, SD 3.82).

Table 1
Number (percentage) of patients whose scores were below cut-off on the face apraxia test and the ideomotor apraxia test

| | Mild AD | Moderate AD | Severe AD |
|---|---|---|---|
| Upper Face Apraxia Test | 4/30 (13%) | 5/19 (26%) | 4/8 (50%) |
| Lower Face Apraxia Test | 4/30 (13%) | 10/19 (53%) | 5/8 (62.5%) |
| Ideomotor Apraxia Test | 4/30 (13%) | 8/19 (42%) | 5/8 (62.5%) |

The severity of cognitive deterioration was evaluated using the M.O.D.A (Milan Overall Dementia Assessment [10]), a neuropsychologically-oriented psychometric severity scale. The M.O.D.A. score ranges from 0 (worst score) to 100 (cut-off score = 85.5, which corresponds to the outer tolerance limit in the normal population). M.O.D.A. raw scores are then adjusted for age and education. M.O.D.A. adjusted scores in this sample ranged from 31.2 to 85.1 (mean 66.99, SD 14.42).

The University of Milan Ethical Committee approved the experiment. All patients and their relatives gave their informed consent to take part in this study.

### *2.2. Experimental design*

A correlative design (Pearson coefficients) was used for most of the analyses in this experiment. A within subject design was adopted for comparing performance on the face apraxia and ideomotor apraxia tests. For this latter comparison a medium effect size ($d = 0.50$) was anticipated for the difference in performance between the two tests. Statistical power with a sample of 57 patients was estimated as 0.96. at $\alpha = 0.05$

### *2.3. Apraxia tests*

#### *2.3.1. Upper and lower face apraxia test*

A test of face apraxia devised, standardised and validated in our laboratory [9,23] was used. The Face Apraxia Test includes two separate subtests suitable for the assessment of movements of the upper and lower face. The Upper Face Apraxia Test includes 9 items (e.g., wrinkle forehead; close eyes; blink left eye), while the Lower Face Apraxia Test incorporates 29 items (e.g., stick out tongue; clear throat; make a high tone). Each item was demonstrated by the examiner and the participant had to respond by imitation immediately after presentation. This procedure was adopted to minimise the confusion between apraxic and verbal comprehension errors. Items were scored as pass or fail. For both subtests a difficulty weighted scoring procedure was employed. The relative difficulty of each item had been previously established in the standardisation phase by rank ordering the test items according to the total number of failures observed in a sample of 180 normal controls. Additionally, each individual score was also adjusted for age and education effects. In the Upper Face Apraxia Test the score ranges from zero to 45.0; the inferential cut-off score, corresponding to the outer tolerance limit is 38.4 while the inner tolerance limit is 42.3. In the Lower Face Apraxia Test the score ranges from zero to 435.0; the cut-off score is 400.0 and the inner tolerance limit is 419.3. Studies on the normative features of the test [9,23] showed that both subtests have an inter-rater reliability of 0.93.

#### *2.3.2. Ideomotor apraxia test*

The test devised by De Renzi et al. [16] was used. This is a 24-item imitation test including an equal number of meaningful and meaningless movements, half requiring the holding of postural gesture and half requiring the execution of a motor sequence. The score for each item ranges from three to zero. A score of three to one is given depending on whether a gesture is performed correctly after the first, second or third attempt. A score of zero is given if the performance is incorrect on all three attempts. The total score ranges from zero to 72. The cut-off score, below which the performance is considered pathological, is 53 [15].

## 3. Results

The results of this study showed that 13/57 (23%) AD patients scored below cut-off on the Upper Face Apraxia test. A higher proportion of them, 19/57 (33%), scored below cut-off on the Lower Face Apraxia test. On the Ideomotor Apraxia test 17/57 (30%) patients had scores which fell below cut-off when their left hand was tested, while a smaller number, 14/57 (24.5%), scored below cut-off when their right hand was tested.

A correlation analysis was run to see whether the AD patients' scores on the Face Apraxia test correlate with disease severity as expressed by M.O.D.A. scores. Results showed that there was a significant correlation between scores on the Upper Face Apraxia test and the M.O.D.A. scores ($r = 0.369$, $p = 0.0045$) and between scores on the Lower Face Apraxia test and the M.O.D.A. scores ($r = 0.537$, $p < 0.0001$). A significant correlation was also present between the M.O.D.A. scores and scores on the Ideomotor Apraxia test with the right ($r = 0.441$, $p = 0.0005$) and left hand ($r = 0.572, p < 0.0001$) respectively. The scores on the Upper Face Apraxia test correlated significantly with the scores on the Ideomotor Apraxia test both with the right ($r = 0.657$, $p < 0.0001$) and the left ($r = 0.622$, $p < 0.0001$) hand. Similarly the scores on the Lower Face Apraxia test correlated significantly with the scores on the Ideomotor Apraxia test both with the right ($r = 0.696$, $p < 0.0001$) and the left ($r = 0.653, p < 0.0001$) hand.

In order to verify whether there was a larger number of patients falling below cut off in the most severe range, the group of patients was divided in three subgroups according to their M.O.D.A. scores. This was done by calculating the difference between the highest and lowest M.O.D.A. score in the group, and by dividing this difference in three equal intervals. Three severity bands were identified; the original patient group was divided in three subgroups labeled mild if their M.O.D.A. score was between 67.1 and 85.1, moderate if their M.O.D.A. score was between 49.1 and 67.0 and severe if their M.O.D.A. score was between 31.2 and 49.0. The percentage of patients below cut-off for each severity band is shown in Table 1.

When a single case analysis was carried out, dissociations emerged. Nine patients showed both apraxia of the upper and lower face. Four had apraxia of the upper face but not of the lower face and 10 showed the converse pattern. As for dissociations between face apraxia and ideomotor apraxia (as expressed by the average score between right and left hand), the analysis showed that nine patients had both upper face apraxia and ideomotor apraxia, while 11 patients had both lower face apraxia and ideomotor apraxia. Of 13 patients with upper face apraxia, four did not have ideomotor apraxia, whereas eight out of 19 patients with lower face apraxia did not have ideomotor apraxia. Only two of those patients with both upper and lower face apraxia did not have also ideomotor apraxia. Only three patients who had ideomotor apraxia showed neither upper nor lower face apraxia. Table 2 provides a summary of the observed associations and dissociations between the three types of apraxia.

Raw scores on both the Upper and Lower Face Apraxia Test were combined together and transformed in percentage of correct score to obtain an index of face

Table 2
Number (percentage) of patients without any form of apraxia and who showed dissociations or associations of upper face apraxia (UFA), lower face apraxia (LFA) and ideomotor apraxia (IMA)

| AD Patients | Number |
|---|---|
| Without any form of apraxia | 31 (54%) |
| With some form of apraxia: | 26 (46%): |
| UFA only | 2 (8%) |
| LFA only | 6 (23%) |
| UFA+LFA only | 2 (8%) |
| IMA only | 3 (11%) |
| UFA+IMA only | 2 (8%) |
| LFA+IMA only | 4 (15%) |
| UFA+LFA+IMA | 7 (27%) |
| **TOTAL** | **57** |

apraxia. Raw scores on the Ideomotor Apraxia Test underwent the same transformation to obtain an index of ideomotor apraxia. The transformation in percentage was necessary to eliminate the difference in scale between the two tests. An analysis of variance was run on the transformed scores. This showed a statistically significant difference between hand and face apraxia ($F_{(1,56)} = 4.742$, $p = 0.0337$) indicating that in this group of AD patients ideomotor apraxia was more severe than face apraxia. The effect size observed was very close ($d = 0.48$) to the one estimated at the inception of the study.

## 4. Discussion

Results from this study show that there is a proportion of AD patients who fail on face apraxia tests and that dissociations between upper and lower face apraxia or between face apraxia and ideomotor apraxia are present in AD.

Rapcsak et al. [55] found that the 28 AD patients they tested failed only 17% of the items of their oral apraxia test (compared to performance at ceiling of 23 healthy controls). These authors interpreted their findings as an indication of relative sparing of face apraxia in AD, which would be justified in terms of anatomical segregation. Studies with patients with focal brain lesions have shown that the brain areas involved in lower face apraxia are the frontal and central opercula, the first temporal convolution and the anterior portion of the insula [63], as well as the striatum [44,54]. These regions are not the most damaged by AD [11]. In contrast to Rapcask et al's findings [55], Taylor [62] reported that 92% (23/25) of his AD patients scored below cut-off in the lower face apraxia test [32] he used. Our data show a different scenario. Both upper and lower face apraxia are found with low frequency (13% for both types of apraxia) in patients in the very mild stage. The frequency of these disorders shows a substantial increase with the progression of the disease moving up to much higher figures (26% and 50% for upper face apraxia and 56% and 62.5% for lower face apraxia) as the disease progresses into moderate and severe stages. The anatomical interpretation proposed by Rapcsak et al. [55] is based on the assumption that the degenerative process in AD does not encroach upon the anterior brain areas responsible for face eupraxia. However, the network involved in face eupraxia might be less localised than previously thought. A recent study confirmed the view that a distributed network in the left hemisphere is involved in face eupraxia and pointed to a role of a similarly distributed network present also in the right hemisphere [9], especially associated with movements of the upper face. The possibility that the right hemisphere plays some role in face eupraxis is supported by evidence of eyelid opening apraxia secondary to right hemisphere infarction [30]. A few studies have also documented that even posterior lesions can cause face apraxia [8,9,27,28]. The notion that the network involved in eupraxia is more diffuse than previously thought is supported by further data gathered in AD patients. Foster et al. [26] reported evidence of right parietal lobe activation detected in AD patients when performing a limb apraxia imitation task. Further evidence of a largely distributed network is provided by the PET findings in a patient with left fronto-parietal dysfunction who showed preserved gesture comprehension [31].

The contrasting findings between studies may be accounted for by differences in severity amongst the patients included. One could assume that the more severe the patients' disease, the more likely it is that the degenerative process involves one or another part of the face eupraxia network. Our data point to this direction. In our sample, the correlation between severity of dementia, as measured by the M.O.D.A., and face apraxia scores accounts for 33% and 52% (upper and lower face apraxia respectively) of the variability, indicating that overall cognitive severity does play a role especially in lower face apraxia. These findings, however, are hard to reconcile with those of Rapcsak et al. [55] since the patients included in their study were also relatively severe: their MMSE scores ranged from two to twenty (mean 11.8). Severity alone cannot, therefore, be the only factor to account for the difference in findings between studies. Especially, severity does neither account for the difference between the results in our study and Rapcsak et al's one [55], nor for the difference between this latter and Taylor's study [62]. A more likely explanation may reside in differences between their psychometric choices. While Taylor [62] based his conclusions on the high number of patients failing the entire task, Rapcsak et al. [55] argued for the preservation of face apraxia in AD on the basis of the total count of items failed by the whole group. Had this latter scoring criterion been used, Taylor's [62] patients would have failed only 13% of the items, proving as "mild" as Rapcsak et al.'s patients. Of course the converse may also be true. Since Rapcsak et al.'s [55] controls performed at ceiling, it is likely that most of their patients would have had scores below cut-off had they used one. One could, therefore, conclude from the available evidence on face apraxia in AD, including the findings of the present study, that, especially in the more severe stage of the disease, there is a significant proportion of AD patients showing face apraxia.

The proportion of AD patients showing face apraxia (23% and 33% for upper and lower face respectively) compares with those having limb ideomotor apraxia, shown by 30% of the patients in the present study. Both tests had to be performed on imitation and all items were intransitive in both cases; a few meaningful gestures were present in both the face and the limb tests. The proportion of AD patients with limb apraxia observed in the present study reflects that observed in previous investigations (e.g. [20]: 30%; [70], sample 1: 45%; [34], 36%). Other researchers however, who also used imitation tests, reported much higher prevalence of ideomotor limb apraxia (e.g. [70], sample 2: 84%; [24], 94%). Heterogeneity amongst different psychometric properties of the various measures of limb apraxia (number of items, selection of cut-off point) used in all different studies may account for part of the discrepancy among investigations.

As for face apraxia, our data show that the proportion of patients with ideomotor apraxia increases significantly with disease severity ranging from 13% in the mild subgroup to 42% in the moderate, to 62.5% in the more severe one. Edwards et al. [25] subdivided AD patients in different stages of dementia severity and reported an increasing frequency of limb apraxia, which ranged from 35 to 98%, as dementia severity increased. Accordingly, Travniczek-Marterer et al. [64] found that performance on ideomotor limb apraxia tests begins to deteriorate in the early stages of the disease. The rate of progression of ideomotor limb apraxia is very slow. Della Sala et al. [20] followed 18 patients with AD longitudinally for seven months and found that only two patients, who were not apraxic on the first instance, performed below cut-off at re-test.

In the present study, face and limb apraxia showed high intercorrelations. In addition to severity, ideomotor apraxia accounts for 68% of the variability on the Upper Face Apraxia test and for 72% of the variability on the Lower Face Apraxia test. Some dissociations were, however, present either between upper and lower face apraxia or between face apraxia (upper, lower or both) and ideomotor apraxia. Dissociations between face apraxia and ideomotor apraxia have been previously reported [6,17,42,45,68]. The presence of dissociations gives the opportunity for cognitive speculations. Among the possible models of apraxia a dichotomy between unitary versus non-unitary models has been postulated [54]. The unitary model would not support the presence of dissociating performance between different types of apraxia, whilst the non-unitary one would account for dissociations. Our findings of several dissociations support a separable model. However, given the frequency of concomitant ideomotor and facial apraxia both in patients with focal brain lesions (e.g. 72% in De Renzi et al.'s sample [17]) and in our own sample of patients with AD, the hypothesis of partially overlapping systems seems the most plausible.

## Acknowledgement

This study was partially supported by 60% MURST 1997 and 1998 grant to HS
